# Maternal environment shapes the life history and susceptibility to malaria of *Anopheles gambiae *mosquitoes

**DOI:** 10.1186/1475-2875-10-382

**Published:** 2011-12-21

**Authors:** Lena M Lorenz, Jacob C Koella

**Affiliations:** 1 Division of Biology, Imperial College London, Silwood Park Campus, Ascot SL5 7PY, UK; 2 Department of Disease Control, Faculty of Infectious and Tropical Diseases, London School of Hygiene and Tropical Medicine, London WC1E 7HT, UK

**Keywords:** Maternal effects, *Anopheles gambiae*, Malaria, Immune priming, Host-parasite relationships

## Abstract

**Background:**

It is becoming generally recognized that an individual's phenotype can be shaped not only by its own genotype and environmental experience, but also by its mother's environment and condition. Maternal environmental factors can influence mosquitoes' population dynamics and susceptibility to malaria, and therefore directly and indirectly the epidemiology of malaria.

**Methods:**

In a full factorial experiment, the effects of two environmental stressors - food availability and infection with the microsporidian parasite *Vavraia culicis *- of female mosquitoes (*Anopheles gambiae *sensu stricto) on their offspring's development, survival and susceptibility to malaria were studied.

**Results:**

The offspring of *A. gambiae s.s*. mothers infected with *V. culicis *developed into adults more slowly than those of uninfected mothers. This effect was exacerbated when mothers were reared on low food. Maternal food availability had no effect on the survival of their offspring up to emergence, and microsporidian infection decreased survival only slightly. Low food availability for mothers increased and *V. culicis*-infection of mothers decreased the likelihood that the offspring fed on malaria-infected blood harboured malaria parasites (but neither maternal treatment influenced their survival up to dissection).

**Conclusions:**

Resource availability and infection with *V. culicis *of *A. gambiae s.s*. mosquitoes not only acted as direct environmental stimuli for changes in the success of one generation, but could also lead to maternal effects. Maternal *V. culicis *infection could make offspring more resistant and less likely to transmit malaria, thus enhancing the efficacy of the microsporidian for the biological control of malaria.

## Background

The population dynamics of anopheline mosquitoes and the epidemiological dynamics of malaria have long been recognized to depend on environmental variables such as temperature [1-3] and its daily variability [4]. In addition to shaping the individuals exposed to them, environmental factors can also have longer-term effects on future generations. It is becoming generally accepted that an individual's phenotype can be influenced not only by its own genotype and environmental experience, but also by its mother's environment and condition [5-7]. Such maternal effects can be adaptive [8,9]. In *Anopheles stephensi*, for example, the daughters of low-food mothers take up more blood and lay more eggs than the daughters of well-fed females, even if the daughters themselves experience the same environment [10]. Alternatively, maternal effects may reflect the mother's condition [11]. Food-deprived mothers may not be able to compensate for their poor environment and, therefore, have offspring of lower quality [12-15]. Thus, the maternal environment could influence the population dynamics of mosquitoes.

An individual's susceptibility to parasites may be influenced by its mother's environmental quality [16], or whether she herself was infected [17-20]. Thus, it is possible, and indeed likely, that malaria transmission could be changed by maternal effects, and that control strategies altering the mosquitoes' environment will affect the main parameters underlying the epidemiology of malaria - the mosquitoes' susceptibility to malaria, longevity and other life-history traits - not only directly, but also indirectly and over a longer period.

Mothers may be exposed to several types of environmental stress simultaneously, including poor resource availability, changing climate, competition with conspecifics, predation and parasitism. Food stress, for example, can exacerbate the harmful effects of infection [21-23], so it may also increase the importance of trans-generational effects. Potentially adaptive effects may also switch with the presence of a second stressor. The consequences of a combination of maternal stressors (such as limited food availability and infection) on the transfer of a female's experiences to her offspring could have important and unexpected consequences for the dynamics of the host and the parasite.

The microsporidian parasite *Vavraia culicis *[24] has been suggested as a potential late-acting control agent of anopheline malaria vectors that will impose little evolutionary pressure for resistance [25,26]. Mosquito larvae orally ingest *V. culicis *spores and become infected; infectivity rates range between 90-100%. Effects of the microsporidian on *Anopheles gambiae sensu stricto (s.s.) *include delayed pupation by 10%, decreased fecundity by 23% and reduced adult lifespan by 27% [25], and reduced susceptibility to malaria [27]. Despite the obvious reductions in fitness, resistance mechanisms of mosquitoes to microsporidians are not known [28].

In this study, maternal effects were evaluated for two environmental variables: the food regime available to larvae and infection by *V. culicis*. Similarly to microsporidian infection, poor nutrition of mosquito larvae increases development time [23,29] and decreases survival [25]. Here, a full factorial experiment where *Anopheles gambiae s.s*. mosquitoes were exposed to a low or high food regime with or without infection with *V. culicis *was conducted. It was investigated how these maternal experimental conditions influence the offspring with regard to susceptibility to malaria, which directly determines malaria transmission, and two life-history traits that influence the transmission indirectly by affecting the mosquitoes' population dynamics (larval survival and developmental time).

## Methods

**Figure 1:**
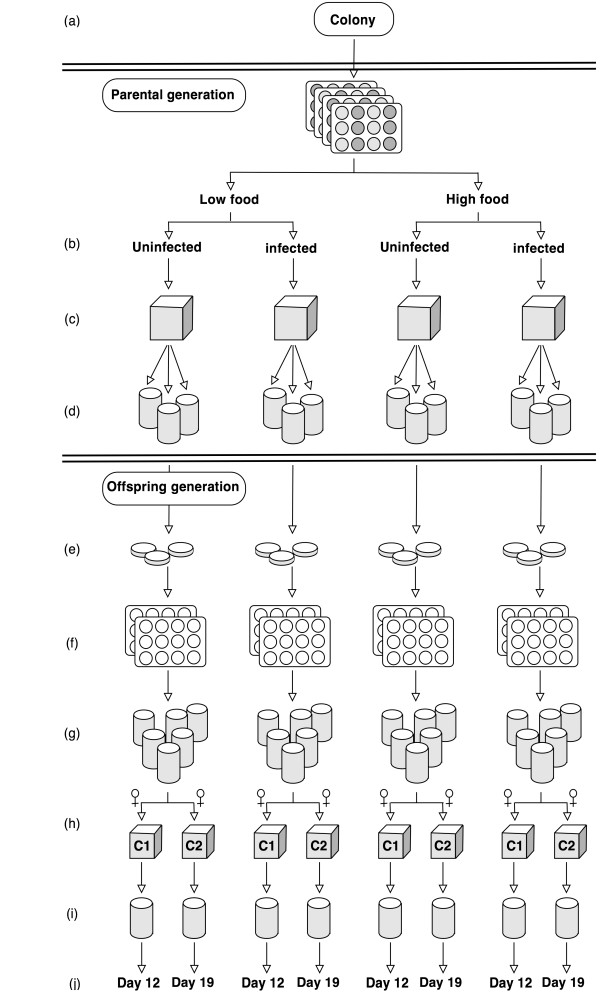
**Schematic representation of the experimental set-up**. For the parental generation, (**a**) 600 *Anopheles gambiae (s.s.) *larvae were reared individually under high and low food conditions, and (**b**) 300 larvae of each food treatment were exposed to *Vavraia culicis *spores. (**c**) After emergence, the females were placed into mating cages according to their treatment, given access to uninfected males, and allowed to blood-feed. (**d**) Fully engorged females (= mothers) were put into individual egg-laying cups. (**e**) To start the offspring generation, the eggs of each mother were bleached and placed into Petri dishes for hatching. (**f**) Six larvae of each mother were reared individually in 12-well plates. (**g**) The pupae were placed into individual tubes for emergence. (**h**) Two adult females of each family were moved to two cages (replicates) per treatment, and allowed to feed on malaria-infectious blood. (**i**) After blood-feeding the mosquitoes were held individually in cups until dissection. (**j**) Mosquitoes of cages C1 were dissected for oocysts 12 days after blood-feeding; the mosquitoes of cages C2 were dissected for sporozoites 19 days after blood-feeding.

Figure 1 shows the experimental design of this study. The mosquitoes originated from a genetically diverse colony established from *A. gambiae s.s*. caught in Yaoundé, Cameroon [30]. Mosquitoes were held at 26 ± 1°C and 70 ± 5% relative humidity with 12 hours light: dark cycles at Silwood Park Campus (Imperial College London, UK). Infection of mosquitoes with *Plasmodium berghei *took place in an insectary kept at 19 ± 1°C and 70 ± 5% relative humidity with 12 hours light: dark cycles. The *Vavraia culicis floridensis *spores were provided to us by J.J. Becnel (USDA Gainesville, USA). At Silwood Park Campus, the microsporidian parasite has been propagated in large groups of *Aedes aegypti *and *A. gambiae s.s*. mosquitoes. For consistency with earlier studies, the parasite is called *V. culicis *throughout the manuscript, whilst acknowledging the subspecies status of the Florida isolate [24].

### Maternal generation

For the maternal generation, 300 larvae were individually reared in 2 ml de-ionized water in 12-well plates for each of four treatment groups: (1) no microsporidian infection, reared on high food (Tetramin fish food: Day 0 (hatching): 0.06 mg, Day 1: 0.12 mg, Day 2: 0.24 mg, Day 3: 0.36 mg, Day 4: 0.48 mg, Day 5 and following days: 0.6 mg per individual); (2) infection with 20,000 *V. culicis *spores, reared on high food; (3) uninfected, reared on low food (half of the high amount of food increasing in incremental steps to account for larval growth; see above); (4) microsporidian-infected, reared on low food (Figure 1a). Food and infection levels were chosen on the basis of past experience: The standard amount generally lets larvae develop within about 8 days from hatching to adult with low levels of juvenile mortality, whereas half of this amount puts larvae under increased nutritional and developmental stress (JCK, *pers. comm*.). An intermediate spore concentration affects the host adversely by delaying pupation and decreasing survival, but without killing mosquitoes too quickly [25] to ensure that enough mothers survived and reproduced despite the harmful fitness effects of the microsporidian. Larvae were fed every 24 hours and those to be infected were exposed to the microsporidian when they were two days old (Figure 1b). Larvae pupated after seven to nine days. Pupae were placed into individual 50 ml Falcon tubes to emerge. For each treatment group, 41-46% emerged as female mosquitoes. All females from one treatment group were placed into one cage the day after they emerged. A mix of 66 males from the two microsporidian-free treatments was added to each one of the four cages one or two days after female emergence (Figure 1c). Adult mosquitoes were provided daily with cotton soaked with 6% glucose solution and were allowed to mate for three days, when females were offered a blood meal on JCK's arm for 10 min. One day after the blood meal, the fully engorged female mosquitoes (*N *= 96) were placed into individual oviposition cups containing a dish lined with filter paper filled with 20 ml de-ionized water (Figure 1d). The eggs from each mother were placed into individual Petri dishes (Figure 1e). Spores of *V. culicis *can be attached to the eggs of microsporidian-infected females [31], though the natural occurrence of the frequency and intensity of the trans-ovarial transmission route has not been quantified. Due to this uncertainty, and in order to disentangle physiological maternal effects from external ones, spores were eliminated by bleaching all eggs (irrespective of maternal infection) with 1% household bleach on the day they were laid [32].

After mothers had laid their eggs, they were killed and stored at -20°C. Subsequently, they were individually homogenised in 0.1 ml of de-ionised water, and *V. culicis *infection was confirmed by counting spores under a phase-contrast microscope (400 × magnification) using a haemacytometer. All 43 mothers from the infection treatments were infected with *V. culicis *spores.

### Offspring generation

For each of the 96 mothers, the susceptibility of her offspring to malaria was investigated. To ensure that each mother was represented equally, one female offspring per mother for each measurement was used. To have a good chance of obtaining the required two females per mother, six larvae per mother were reared. If more than two females survived to adulthood, two were chosen haphazardly. Life-history traits (larval survival and age at pupation) were measured for each of the six larvae.

The larvae were reared individually on the high food level (see above; Figure 1f). Larval mortality and pupation were checked every 24 hours. Pupae were placed into individual 50 ml Falcon tubes and allowed to emerge (Figure 1g). Two cages for each of the four maternal treatments were prepared, each containing one of the two female offspring haphazardly selected per mother to measure two aspects of susceptibility: the parasite's development to 1) the oocyst stage and 2) the sporozoite stage. One of the cages later gave the mosquitoes for oocyst detection; the other the mosquitoes for sporozoite-detection (Figure 1h).

GFP-expressing transgenic *P. berghei *ookinetes (PbGFP_CON _strain; [33,34]) were produced by R. Armson at R.E. Sinden's laboratory at Imperial College London according to the laboratory's standard protocol. The ookinete culture was centrifuged at 500 g for 10 minutes at 19°C, the supernatant was removed and the ookinetes were counted under a microscope (400 × magnification) with a haemacytometer. Blood of uninfected mice was added to give a concentration of 800 ookinetes per μl. 400 μl of the mixture were injected into membrane feeders that had been preheated to 37 ± 1°C with a water bath and covered with Parafilm "M" (Pechiney Plastic Packaging). The mosquitoes had access to glucose up to 24 hours before their blood meal. They were blood-fed with the malaria-infectious blood meal for one hour in darkness at 19 ± 1°C five to seven days after emerging. Each group of mosquitoes (two cages per maternal treatment) was provided with two membrane feeders to reduce the effect of possible differences among feeders. One day after the blood meal, fully engorged mosquitoes were placed into cups, which were kept at 19 ± 1°C and were supplied with cotton soaked with 6% glucose solution every 24 hours until dissection (Figure 1i). Twelve days after the blood meal, the mosquitoes from one of the cages from each treatment were dissected (*N *= 42) and their midguts fixed with 4% formaldehyde in PBS and mounted in antifade mounting fluid (Vectashield, Vector Laboratories Inc., Burlingame). Oocysts on midguts were counted under a fluorescent microscope (100 × magnification). Nineteen days after the blood meal, the mosquitoes from the second cages were dissected (*N *= 24), their salivary glands mounted on slides and their sporozoites counted under a microscope (400 × magnification). The dissection days were chosen to be 12 and 19 days after the blood meal in order to maximize the malaria parasite detection rate (E. Dawes, *pers. comm*.; Figure 1j).

### Statistical analysis

Full models included maternal food level, maternal microsporidian infection status, their interaction and (when necessary) mother as a random factor. The final models were selected by comparing Akaike Information Criterion (AIC) values, with models with the lowest AIC values chosen as the minimum models [35]. The significance level α was set to 0.05.

The number of eggs produced by mothers from the four treatment groups was analysed with an analysis of variance. To test whether maternal treatment had any effects on the egg hatching rate, a generalized linear model (GLM) with binomial error structure and logit link function was performed.

As the offspring's age at pupation was restricted to seven to nine days after hatching (with most pupating after seven or eight days), mosquitoes with early (pupation on day 7) and late (pupation after day 7) pupation were compared. Generalized linear mixed models with binomial error structures and logit link functions were performed to test whether maternal treatments had effects on offspring survival and pupation. 'Mother' was set as the random factor. When testing for effects on pupation, offspring sex was included as a fixed factor in the analysis.

Offspring mosquitoes from each maternal treatment group were reared in two cages and the mosquitoes from each cage were dissected for malaria at different days (12 and 19 days after the blood meal). In order to determine whether maternal treatment had an effect on the proportion of offspring harbouring malaria parasites whilst accounting for survival until the different dissection days, the following two analyses were performed. First, a GLM with binomial error structure and logit link function was fitted to explain the proportion of mosquitoes surviving until the day of dissection. Cage, maternal food, microsporidian infection and their interactions were set as explanatory variables. Second, a GLM with binomial error structure and logit link function with cage, maternal food, microsporidian infection and their interactions was fitted to explain their effects on the proportion of mosquitoes infected with malaria parasites (oocysts or sporozoites).

Analyses were performed with JMP 8 [36] and package 'lme4' [37] in R Version 2.13.0 [38].

## Results

### Maternal traits

*Anopheles gambiae (s.s.) *females reared on high larval food in a microsporidian-free environment laid more than twice as many eggs (58 ± 3 s.e.) as uninfected females reared on low food (27 ± 3 s.e.; F_1,101 _= 40.6, *p *< 0.001). Infection with *V. culicis *reduced the number of eggs laid to 26 (± 3 s.e.) and 19 (± 3 s.e.) for high and low food treatments respectively (F_1,101 _= 44.5, *p *< 0.001). Infection had a larger effect on the total number of eggs produced by well-fed mothers than on those from low-food mothers (54% egg reduction for high-food mothers, 30% reduction for low-food mothers; food by infection: F_1,101 _= 15.6, *p *= 0.001). Of those eggs laid by low food mothers, 75% (C.I. 52%-89%) hatched; 86% (C.I. 72%-94%) from high-food mothers did (χ_1_^2 ^= 17.1, *p *< 0.001). Microsporidian infection reduced the proportion of eggs that hatched from 86% (C.I. 71%-94%) to 74% (C.I. 51%-89%; χ_1_^2 ^= 17.6, *p *< 0.001), but this was not affected by the mother's food level (non-significant interaction term).

### Offspring development

It was tested whether the maternal treatments had an effect on their offspring's larval survival and developmental time. From 96 mothers, 576 offspring were analysed for larval emergence and 552 individuals for pupation. The offspring of *V. culicis*-infected mosquitoes were 3% less likely to survive their juvenile period than those of uninfected mothers (probability of survival of the offspring of uninfected mothers: 97% (C.I. 95%-99%), of infected mothers: 94% (C.I. 90%-96%); χ_1_^2 ^= 3.6, *p *= 0.056). Maternal food had no effect on the offspring's probability of survival (χ_1_^2 ^= 0.2, *p *= 0.70), regardless of the mother's infection status (χ_1_^2 ^= 1.2, *p *= 0.26).

**Figure 2:**
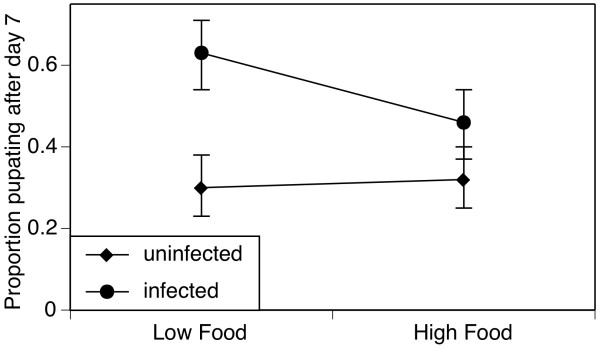
**Maternal effects on offspring development time**. Proportion of *Anopheles gambiae (s.s.) *pupating later than seven days after hatching as a function of their mother's access to food and infection by *Vavraia culicis*. The symbols (diamonds: uninfected mothers; squares: microsporidian-infected mothers) show the proportions, the vertical lines the 95% confidence intervals based on a binomial distribution.

As most individuals pupated seven or eight days after hatching, with only 2.2% pupating later, mosquitoes with early (pupation on day 7) and late (pupation after day 7) pupation were compared. Females pupated significantly later than males (χ_1_^2 ^= 60.3, *p *< 0.001). Maternal microsporidian infection increased the proportion of mosquitoes pupating late from 31% (C.I. 26%-37%) to 54% (C.I. 48%-60%; χ_1_^2 ^= 21.0, *p *< 0.001). When mothers were uninfected, their offspring's development period was not affected by material larval food treatment (χ_1_^2 ^= 2.2, *p *= 0.14). However, in the presence of *V. culicis*, the offspring of mothers exposed to low food availability pupated late (63%, C.I. 54%-71%), while most of the offspring from mothers exposed to abundant food had already pupated by day 7 (Figure 2; food by inflection: χ_1_^2 ^= 6.1, *p *= 0.014).

### Offspring malaria infection

The effects of maternal food and microsporidian infection on the offspring's susceptibility to malaria, measured as the probability that the mosquitoes harboured *Plasmodium *parasites after feeding on an infected blood meal, were investigated. As neither maternal food (χ_1_^2 ^= 0.9, *p *= 0.35) nor maternal *V. culicis *infection (χ_1_^2 ^= 0.1, *p *= 0.92) had an effect on the survival of offspring (*N *= 121) up to their day of dissection, and the two cages had similar levels of malaria infection (χ_1_^2 ^= 0.1, *p *= 0.75), the use of the two cages as replicates to compare malaria-infected with malaria-uninfected mosquitoes was justified.

**Figure 3:**
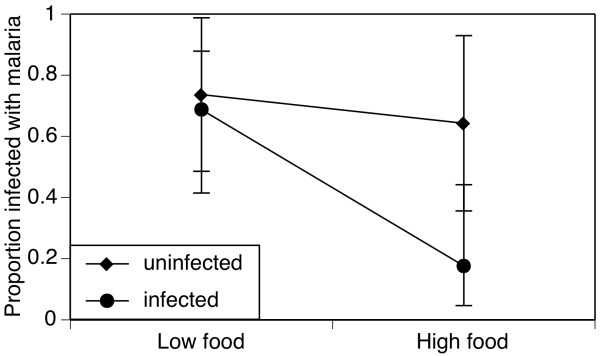
**Maternal effects on offspring malaria infection**. Proportion of females infected with *Plasmodium berghei *parasites as a function of their mothers' access to food and infection by *Vavraia culicis*. The symbols (diamonds: uninfected mothers; squares: microsporidian-infected mothers) show the proportions, the vertical lines the 95% confidence intervals based on a binomial distribution.

66 individuals for maternal effects on offspring malaria infection were analysed. Both maternal treatments affected the success of malaria. The offspring of low-food mothers were, on average, 32% more likely to harbour malaria parasites than those of well-fed females (71%, C.I. 53%-85% vs. 39%, C.I. 22%-58%; χ_1_^2 ^= 6.4, *p *= 0.012). Seventy percent (C.I. 51%-84%) of the offspring of microsporidian-free mothers were infected with *P. berghei*, but only 42% (C.I. 26%-61%) of *V. culicis*-infected females were (χ_1_^2 ^= 4.6, *p *= 0.032). The offspring of high-food, microsporidian-infected mothers were least likely to be infected with malaria parasites (18%, C.I. 5%-44%) whereas the offspring of the other maternal treatments were more susceptible (offspring of high-food, uninfected mothers: 64% (C.I. 36%-86%), of low-food, uninfected mothers: 75% (C.I. 49%-90%), of low-food, infected mothers: 69% (C.I. 41%-88%); Figure 3). However, this interaction was not statistically significant (food-by-infection: χ_1_^2 ^= 2.9, *p *= 0.089).

## Discussion

The two types of maternal stress - low food and infection by *V. culicis *- considered here had different effects on traits that influence the epidemiology of malaria. With both stressors, *A*. *gambiae s.s*. had offspring that took longer to pupate. Offspring were more likely to be infected by *P. berghei *if their mothers were reared on low-food than on high food, whereas offspring from microsporidian-infected mothers were less, rather than more, susceptible to infection by malaria.

### Offspring development

Mothers reared in stressful environments often produce smaller and less viable eggs [13,39,40]; examples show that maternal stress leads to weaker offspring are rove beetles (*Tachyporus hypnorum*, [13]), Hawaiian fruit flies (*Drosophila grimshawi*, [12]) and locusts (*Schistocerca gregaria*, [14]). Infection by the microsporidian *V. culicis *corroborates this pattern: infected *Ae. aegypti *mosquitoes lay smaller eggs (S. Fellous, *pers. comm*), and here, microsporidian-infected mothers laid fewer eggs that were less likely to hatch. The larvae that did hatch were less likely to survive as juveniles and pupated later than the offspring of uninfected mothers. This effect of microsporidian infection was exacerbated when mothers were reared on low food (Figure 2). However, daughters of badly nourished *A. stephensi *females take larger blood meals and lay more eggs than those of well-fed mothers [10], suggesting that offspring could compensate for expected decreased lifespan in response to poor maternal environments [8,41]. Despite clear maternal effects, it is thus not yet clear how maternal stress would affect the long-term population dynamics of the mosquito.

### Offspring malaria infection

Whereas low-food *Daphnia magna *females produce offspring that are less susceptible to bacterial infection than well-fed females [16], daughters of low-food *A. gambiae s.s*. in this experiment were more likely to be infected with malaria. A possible explanation for this result is that stressed mothers cannot compensate for their poor environment, and therefore invest fewer resources in their offspring. This would reduce the offspring's ability to mount costly immune responses [14] and lead to greater susceptibility to infection.

In contrast, maternal infection by *V. culicis *led to offspring that were less likely to harbour malaria (Figure 3). This could be due to trans-generational immune-priming, which occurs in shrimps (*Penaeus monodon*, [42]), water fleas (*D. magna*, [43]), bumblebees (*Bombus terrestris*, [19,44,45]) and yellow mealworm beetles (*Tenebrio molitor*, [17,46,47]). Although immune-priming is usually specific to the parasite species or strain, immune responses of *A. gambiae s.s*. against malaria can be activated with bacterial challenges [48-55]. Microsporidian infection impedes the development of malaria [27,56-60], suggesting that an unspecific component of immune-activation [61] may be extended to trans-generational immune priming of the offspring. Alternatively, the pattern could reflect a trade-off between growth and resistance to infection [62-65]; mothers could subtly change the development of their offspring so that offspring invest more in their immune system at a cost to their growth. For example, daughters of stressed *D*. *magna *mothers increase their growth rates, but are slightly more susceptible to bacterial infection [15]. Trans-generational immune priming in *T. molitor*, on the other hand, resulted in offspring with longer larval development times [17,47]. In this study, offspring from *V. culicis*-infected *A. gambiae s.s*. mothers also took longer to develop into adult mosquitoes, but were less likely to be infected with malaria, thus corroborating a previously demonstrated link between age at pupation and immuno-competence [63]. This association could not explicitly be tested, as all the mosquitoes were pooled after emergence irrespective of their age at pupation.

A lack of sufficient replication limited the power of this study design; each treatment group and sampling of malaria infection was represented by only one mosquito cage. Therefore, maternal treatment, time of dissection and cage were not completely independent variables. However, the use of the two cages as replicates for maternal treatment was still justified as maternal treatments had no effect on offspring survival (maternal food: *p *= 0.35; maternal infection: *p *= 0.92), and malaria infection was similar in both cages (*p *= 0.75). These results add to the confidence that the apparent differences between traits of offspring from different maternal treatment groups represent real differences and trans-generational effects rather than artefacts of cage-effects.

## Conclusions

In summary, resource availability and infection with *V. culicis *of *A. gambiae s.s*. mosquitoes not only act as direct environmental stimuli for changes in the success of one generation, but can also lead to maternal effects. As *A. gambiae s.s*. is a major vector of malaria in sub-Saharan Africa, and microsporidia, such as *V. culicis *and other biopesticides are being considered as potential control agents against this deadly disease [25,66,67], these results may have important social implications. Co-infection of mosquitoes with microsporidia and malaria directly inhibits malaria development [27,59,60]. Maternal *V. culicis *infection could also make offspring more resistant and less likely to transmit malaria, thus further enhancing the efficacy of the microsporidian as a control agent.

## Competing interests

The authors declare that they have no competing interests.

## Authors' contributions

LML and JCK conceived and designed the study. LML carried out the laboratory experiments and performed the statistical analysis. LML and JCK wrote the manuscript. Both authors read and approved the final manuscript.

## References

[B1] Rogers DJ Randolph SE The global spread of malaria in a future, warmer world Science 2000 289 1763 1766 10976072 10.1126/science.289.5485.1763

[B2] Pascual M Ahumada JA Chaves LF Rodo X Bouma M Malaria resurgence in the East African highlands: Temperature trends revisited Proc Natl Acad Sci USA 2006 103 5829 5834 10.1073/pnas.0508929103 16571662 PMC1416896

[B3] Gilioli G Mariani L Sensitivity of *Anopheles gambiae *population dynamics to meteo-hydrological variability: a mechanistic approach Malar J 2011 10 294 10.1186/1475-2875-10-294 21985188 PMC3206495

[B4] Paaijmans KP Blanford S Bell AS Blanford JI Read AF Thomas MB Influence of climate on malaria transmission depends on daily temperature variation Proc Natl Acad Sci USA 2010 107 15135 15139 10.1073/pnas.1006422107 20696913 PMC2930540

[B5] Mousseau TA Dingle H Maternal effects in insect life histories Annu Rev Entomol 1991 36 511 534 10.1146/annurev.en.36.010191.002455

[B6] Bernardo J Maternal effects in animal ecology Am Zool 1996 36 83 105

[B7] Otti O Sadd BM Parental guidance? Trans-generational influences on offspring life history in mosquitoes Trends Parasitol 2008 24 197 199 10.1016/j.pt.2008.02.004 18406210

[B8] Mousseau TA Fox CW The adaptive significance of maternal effects Trends Ecol Evol 1998 13 403 407 10.1016/S0169-5347(98)01472-4 21238360

[B9] Prasad NG Shakarad M Rajamani M Joshi A Interaction between the effects of maternal and larval levels of nutrition on pre-adult survival in *Drosophila melanogaster* Evol Ecol Res 2003 5 903 911

[B10] Grech K Maung LA Read AF The effect of parental rearing conditions on offspring life history in *Anopheles stephensi* Malaria J 2007 6 130139 10.1186/1475-2875-6-130 PMC2034587 17892562

[B11] Rossiter MC Incidence and consequences of inherited environmental effects Annu Rev Ecol Syst 1996 27 451 476 10.1146/annurev.ecolsys.27.1.451

[B12] Jones TM Widemo F Survival and reproduction when food is scarce: Implications for a lekking Hawaiian *Drosophila* Ecol Entomol 2005 30 397 405 10.1111/j.0307-6946.2005.00705.x

[B13] Kyneb A Toft S Effects of maternal diet quality on offspring performance in the rove beetle *Tachyporus hypnorum* Ecol Entomol 2006 31 322 330 10.1111/j.1365-2311.2006.00775.x

[B14] Miller GA Pell JK Simpson SJ Crowded locusts produce hatchlings vulnerable to fungal attack Biol Letters 2009 5 845 848 10.1098/rsbl.2009.0495 PMC2828005 19675004

[B15] Frost PC Ebert D Larson JH Marcus MA Wagner ND Zalewski A Transgenerational effects of poor elemental food quality on *Daphnia magna* Oecologia 2010 162 865 872 10.1007/s00442-009-1517-4 19957090

[B16] Mitchell SE Read AF Poor maternal environment enhances offspring disease resistance in an invertebrate Proc R Soc London, B 2005 272 2601 2607 10.1098/rspb.2005.3253 PMC1559984 16321782

[B17] Roth O Joop G Eggert H Hilbert J Daniel J Schmid-Hempel P Kurtz J Paternally derived immune priming for offspring in the red flour beetle, *Tribolium castaneum* J Anim Ecol 2010 79 403 413 10.1111/j.1365-2656.2009.01617.x 19840170

[B18] Little TJ Kraaijeveld AR Ecological and evolutionary implications of immunological priming in invertebrates Trends Ecol Evol 2004 19 58 60 10.1016/j.tree.2003.11.011 16701227

[B19] Moret Y Schmid-Hempel P Entomology - Immune defence in bumble-bee offspring Nature 2001 414 506 506 11734840 10.1038/35107138

[B20] Freitak D Heckel DG Vogel H Dietary-dependent trans-generational immune priming in an insect herbivore Proc R Soc London, B 2009 276 2617 2624 10.1098/rspb.2009.0323 PMC2686660 19369263

[B21] Brown MJF Loosli R Schmid-Hempel P Condition-dependent expression of virulence in a trypanosome infecting bumblebees Oikos 2000 91 421 427 10.1034/j.1600-0706.2000.910302.x

[B22] Bedhomme S Agnew P Sidobre C Michalakis Y Virulence reaction norms across a food gradient Proc R Soc London, B 2004 271 739 744 10.1098/rspb.2003.2657 PMC1691653 15209108

[B23] Fellous S Koella JC Cost of co-infection controlled by infectious dose combinations and food availability Oecologia 2010 162 935 940 10.1007/s00442-009-1535-2 20033214

[B24] Vàvra J Becnel JJ *Vavraia culicis *(Weiser, 1947) Weiser, 1977 revisited: Cytological characterisation of a *Vavraia culicis*-like microsporidium isolated from mosquitoes in Florida and the establishment of *Vavraia culicis floridensis *subsp n Folia Parasitol (Praha) 2007 54 259 271 18303767

[B25] Lorenz LM Koella JC The microsporidian parasite *Vavraia culicis *as a potential late-life acting control agent of malaria Evol Appl 2011 4 783 790 10.1111/j.1752-4571.2011.00199.x PMC3352544 25568022

[B26] Koella JC Lynch PA Thomas MB Read AF Towards evolution-proof malaria control with insecticides Evol Appl 2009 2 469 480 10.1111/j.1752-4571.2009.00072.x PMC3352447 25567892

[B27] Bargielowski I Koella JC A possible mechanism for the suppression of *Plasmodium berghei *development in the mosquito *Anopheles gambiae *by the microsporidian *Vavraia culicis* PLoS One 2009 4 e4676 10.1371/journal.pone.0004676 19277119 PMC2651578

[B28] Agnew P Becnel JJ Ebert D Michalakis Y Bourtzis K, Miller T Symbiosis of microsporidia and insects Insect Symbiosis 2003 2 Boca Raton, FL: CRC Press 145 163

[B29] Bradshaw WE Johnson K Initiation of metamorphosis in the pitcher-plant mosquito: Effects of larval growth history 1995 76 2055 2065

[B30] Mendes AM Schlegelmilch T Cohuet A Awono-Ambene P De Iorio M Fontenille D Morlais I Christophides GK Kafatos FC Vlachou D Conserved mosquito-parasite interactions affect development of *Plasmodium falciparum *in Africa PLoS Pathog 2008 4 e1000069 10.1371/journal.ppat.1000069 18483558 PMC2373770

[B31] Andreadis TG Microsporidian parasites of mosquitoes J Am Mosq Control Assoc 2007 23 3 29 10.2987/8756-971X(2007)23[3:MPOM]2.0.CO;2 17853594

[B32] MR4 Methods in Anopheles Research http://www.mr4.org/Portals/3/Methods_in_Anopheles_Research.pdf

[B33] Franke-Fayard B Trueman H Ramesar J Mendoza J van der Keur M van der Linden R Sinden RE Waters AP Janse CJ A *Plasmodium berghei *reference line that constitutively expresses GFP at a high level throughout the complete life cycle Mol Biochem Parasitol 2004 137 23 33 10.1016/j.molbiopara.2004.04.007 15279948

[B34] Janse CJ Franke-Fayard B Mair GR Ramesar J Thiel C Engelmann S Matuschewski K van Gemert GJ Sauerwein RW Waters AP High efficiency transfection of *Plasmodium berghei *facilitates novel selection procedures Mol Biochem Parasitol 2006 145 60 70 10.1016/j.molbiopara.2005.09.007 16242190

[B35] Akaike H Petrov BN, Csaki F Information theory as an extension of the maximum likelihood principle Second international symposium on information theory 1973 Budapest, Hungary: Akademiai Kiado 267 281

[B36] SAS Institute Inc JMP 8 Cary, NC 1989 2008

[B37] Bates D Maechler M Bolker B lme4: Linear mixed-effects models using S4 classes http://CRAN.R-project.org/package=lme4

[B38] R Development Core Team R: A language and environment for statistical computing http://www.R-project.org

[B39] Fox CW Czesak ME Evolutionary ecology of progeny size in arthropods Ann Rev Entomol 2000 45 341 369 10.1146/annurev.ento.45.1.341 10761581

[B40] Gliwicz ZM Guisande C Family-planning in *Daphnia*: Resistance to starvation in offspring born to mothers grown at different food levels Oecologia 1992 91 463 467 10.1007/BF00650317 28313496

[B41] Mousseau TA Fox CW Maternal effects as adaptations 1998 Oxford: Oxford University Press

[B42] Huang CC Song YL Maternal transmission of immunity to white spot syndrome associated virus (WSSV) in shrimp (*Penaeus monodon*) Dev Comp Immunol 1999 23 545 552 10.1016/S0145-305X(99)00038-5 10579383

[B43] Little TJ O'Connor B Colegrave N Watt K Read AF Maternal transfer of strain-specific immunity in an invertebrate Curr Biol 2003 13 489 492 10.1016/S0960-9822(03)00163-5 12646131

[B44] Sadd BM Kleinlogel Y Schmid-Hempel R Schmid-Hempel P Trans-generational immune priming in a social insect Biol Letters 2005 1 386 388 10.1098/rsbl.2005.0369 PMC1626361 17148213

[B45] Sadd BM Schmid-Hempel P Facultative but persistent transgenerational immunity via the mother's eggs in bumblebees Curr Biol 2007 17 R1046 R1047 10.1016/j.cub.2007.11.007 18088585

[B46] Moret Y 'Trans-generational immune priming': specific enhancement of the antimicrobial immune response in the mealworm beetle, *Tenebrio molitor* Proc R Soc London, B 2006 273 1399 1405 10.1098/rspb.2006.3465 PMC1560290 16777729

[B47] Zanchi C Troussard JP Martinaud G Moreau J Moret Y Differential expression and costs between maternally and paternally derived immune priming for offspring in an insect J Anim Ecol 2011 80 1174 1183 10.1111/j.1365-2656.2011.01872.x 21644979

[B48] Dimopoulos G Richman A Muller HM Kafatos FC Molecular immune responses of the mosquito *Anopheles gambiae *to bacteria and malaria parasites Proc Natl Acad Sci USA 1997 94 11508 11513 10.1073/pnas.94.21.11508 9326640 PMC23521

[B49] Richman AM Dimopoulos G Seeley D Kafatos FC *Plasmodium *activates the innate immune response of *Anopheles gambiae *mosquitoes EMBO J 1997 16 6114 6119 10.1093/emboj/16.20.6114 9321391 PMC1326295

[B50] Lowenberger CA Kamal S Chiles J Paskewitz S Bulet P Hoffmann JA Christensen BM Mosquito-*Plasmodium *interactions in response to immune activation of the vector Exp Parasitol 1999 91 59 69 10.1006/expr.1999.4350 9920043

[B51] Dimopoulos G Christophides GK Meister S Schultz J White KP Barillas-Mury C Kafatos FC Genome expression analysis of *Anopheles gambiae*: Responses to injury, bacterial challenge, and malaria infection Proc Natl Acad Sci USA 2002 99 8814 8819 10.1073/pnas.092274999 12077297 PMC124381

[B52] Dong YM Aguilar R Xi ZY Warr E Mongin E Dimopoulos G *Anopheles gambiae *immune responses to human and rodent *Plasmodium *parasite species PLoS Pathog 2006 2 513 525 10.1371/journal.ppat.0020052 PMC1475661 16789837

[B53] Dong Y Manfredini F Dimopoulos G Implication of the mosquito midgut microbiota in the defense against malaria parasites PLoS Pathog 2009 5 e1000423 10.1371/journal.ppat.1000423 19424427 PMC2673032

[B54] Dong YM Dimopoulos G *Anopheles *fibrinogen-related proteins provide expanded pattern recognition capacity against bacteria and malaria parasites J Biol Chem 2009 284 9835 9844 10.1074/jbc.M807084200 19193639 PMC2665105

[B55] Meister S Agianian B Turlure F Relogio A Morlais I Kafatos FC Christophides GK *Anopheles gambiae *PGRPLC-mediated defense against bacteria modulates infections with malaria parasites PLoS Pathog 2009 5 e1000542 10.1371/journal.ppat.1000542 19662170 PMC2715215

[B56] Fox RM Weiser J A microsporidian parasite of *Anopheles gambiae *in Liberia J Parasitol 1959 45 21 30 10.2307/3274782 13631568

[B57] Hulls RH The adverse effects of a microsporidian on sporogony and infectivity of *Plasmodium berghei* Trans R Soc Trop Med Hyg 1971 65 421 422 4398586 10.1016/0035-9203(71)90120-9

[B58] Gajanana A Tewari SC Reuben R Rajagopalan PK Partial suppression of malaria parasites in *Aedes aegypti *and *Anopheles stephensi *doubly infected with *Nosema algerae *and *Plasmodium* Indian J Med Res 1979 70 417 423 395108

[B59] Margos G Maier WA Seitz HM The effect of nosematosis on the development of *Plasmodium falciparum *in *Anopheles stephensi* Parasitol Res 1992 78 168 171 10.1007/BF00931661 1557330

[B60] Schenker W Maier WA Seitz HM The effects of *Nosema algerae *on the development of *Plasmodium yoelii nigeriensis *in *Anopheles stephensi* Parasitol Res 1992 78 56 59 10.1007/BF00936182 1584748

[B61] Biron DG Agnew P Marche L Renault L Sidobre C Michalakis Y Proteome of *Aedes aegypti *larvae in response to infection by the intracellular parasite *Vavraia culicis* Int J Parasitol 2005 35 1385 1397 10.1016/j.ijpara.2005.05.015 16102770

[B62] Boots M Begon M Trade-offs with resistance to a granulosis-virus in the Indian meal moth, examined by a laboratory evolution experiment Funct Ecol 1993 7 528 534 10.2307/2390128

[B63] Koella JC Boëte C A genetic correlation between age at pupation and melanization immune response of the yellow fever mosquito *Aedes aegypti* Evolution 2002 56 1074 1079 12093022 10.1111/j.0014-3820.2002.tb01419.x

[B64] Rantala MJ Roff DA An analysis of trade-offs in immune function, body size and development time in the Mediterranean field cricket, *Gryllus bimaculatus* Funct Ecol 2005 19 323 330 10.1111/j.1365-2435.2005.00979.x

[B65] Mooney KA Halitschke R Kessler A Agrawal AA Evolutionary trade-offs in plants mediate the strength of trophic cascades Science 2010 327 1642 1644 10.1126/science.1184814 20339073

[B66] Thomas MB Read AF Can fungal biopesticides control malaria? Nat Rev Microbiol 2007 5 377 383 10.1038/nrmicro1638 17426726

[B67] Koella JC Lorenz L Bargielowski I Microsporidians as evolution-proof agents of malaria control? Adv Parasitol 2009 68 315 327 19289199 10.1016/S0065-308X(08)00612-X

